# Weight underestimation and high cardiovascular disease risk among South African adults with obesity: implications for integrated obesity prevention

**DOI:** 10.1186/s12889-025-23378-9

**Published:** 2025-06-04

**Authors:** Kufre Joseph Okop, Yusuf Amuda Agabi, Victoria Joseph

**Affiliations:** 1 Chronic Disease Initiative for Africa (CDIA), Department of Medicine, University of Cape Town, Cape Town, South Africa; 2 Department of Prevention and Evaluation, Leibniz institute for Prevention Research and Epidemiology - BIPS, Bremen, Germany; 3 Citizen Science Research Foundation (CSRF), Cape Town, South Africa; 4 Department of Microbiology, University of Jos, Jos, Nigeria

**Keywords:** Weight underestimation, Body image, Cardiovascular disease risk, South African, Obesity, Integrated prevention

## Abstract

**Background:**

The contribution of body size perception to cardiovascular disease risk among persons with inherent negative body image perceptions in African settings has not been established. This study describes body image, weight discordance and absolute 10-year CVD risk score among predominantly obese black South African adults.

**Methods:**

A cohort study involving 920 adults aged 35–78 years in an urban township, a rural community, and South Africa. Medical history, anthropometrics and blood pressure were taken at baseline and follow-up. Body image perceptions were obtained using a validated body shape questionnaire at follow-up, and each participant’s absolute 10-year CVD risk scores were also determined. Descriptive and mean comparison analyses were undertaken using SPSS version 26.

**Results:**

A higher proportion of women (84.1%) compared to men (32.2%) were overweight (BMI > 25 kg/m^2^). Increasing weight underestimation was associated with relative weight gain in both genders. Body weight underestimation had a weak, significant association with 10-year absolute CVD risk scores. About a quarter of men, compared to 58.3% of women, 42% of those with normal weight, and 30% with obesity, had a ‘high’ 10-year CVD risk score (i.e. score ≥ 20%). In both the urban (62% vs. 30%) and the rural (53% vs. 20%) communities, men had higher CVD risk scores than women, and these comparisons were statistically significant (*p* < 0.05).

**Conclusion:**

Obesity and CVD risk prevention programmes targeting black South Africans should consider a sustained healthy weight maintenance intervention focusing on personalised self-assessments of weight gain intentions and body size preferences.

## Introduction

The burden of cardiovascular disease (CVD) among adults in sub-Saharan Africa populations and globally increases as obesity rates rises [1–3]. In South Africa, with inherent socio-economic inequalities, overweight/obesity rate among women is rising disproportionately (68%) compared to men (31%) [4, 5]. Obesity considered an independent risk factor for CVD, the leading cause of death in South African adults aged 25–49 years [6].

Body image perception is regarded as an important contributor to the obesity epidemic and health risk including CVD risk among African [7–9]. Evidence indicates that body image perceptions can impact on the modifiable CVD risk factors, particularly, among adults and adolescents in many populations [10–12], and ultimately, CVD prevalence. A recent study involving 6479 adults reported that women who overestimated their body sizes had higher prevalence and risk of metabolic syndrome, whereas men who underestimated their body sizes had lower prevalence and risk of metabolic syndrome [13].

The perception of one’s own body size, particularly, underestimation of weight has been linked with CVD risk factors such as obesity, poor intake of fruits and vegetables, physical inactivity among others in African populations [14, 15]. It is also confirmed by earlier studies that body image perception has a profound influence on many CVD risk factors in settings where large body size is perceived as a sign of quality of life [16]. Particularly, among black Africans, it is also known that distorted body image perception is associated with weight gain, inactivity, adverse eating behaviours, sustained obesity, and increasing eating disorders [17, 18]. A study also reported a possible link between personal perception of the threat of CVD risk and body image in adults [19]. Our earlier study among black South Africans with obesity had reported high rate of weight underestimated (85%) [9]. In that study, those who perceived CVD threat, compared with those who did not, were more likely to be dissatisfied with their body sizes.

Furthermore, there are indications of positive association between body image perceptions, CVD risk factors and behaviours such as smoking, nutrition and psychological disorders. This is confirmed by earlier studies that reported increasing smoking pattern, alcohol use and health disorders among Africans and African immigrants in Europe with body image discrepancies [20, 21]. In addition, psychological effects such as anxiety, depression, and stress, as well as substance abuse have been associated with body image distortions among black Africans (6). The effect of body image is not only seen among Africans, it is however, also reported among adults and young persons in other populations. In Hispanic women and African Americans, for example, body image dissatisfaction was associated with negative eating behaviours [17], and among Malaysians adolescents [22], it was associated with low physical activity. Among Spanish adolescent, body image was associated with mental health symptoms [23].

Total (or absolute) CVD risk score is usually measured using Framingham-based risk score (FRS), and has been validated for Africans and used to efficiently identify persons at high risk of CVD [24, 25]. Specifically, the absolute 10-year risk score for CVD has also been validated in a large and diverse populations in South Africa [25, 26], and used in primary care and shown to be a cost-efficient strategy of identifying persons at risk of CVD [27–29].

The causal correlation between CVD mortality risk and body image has not been fully established. However, preliminary results from previous study among adult black South Africans indicated body image perceptions was associated with low willingness to lose weight among those persons with obesity at various range of 10-year CVD risk score [30, 31]. This study hypothesized that body image perception, particularly, weight underestimation is associated with weight gain, and invariably can affect total 10-year CVD risk score among predominantly obese persons. This study, therefore, explores the weight discordance in relation to 10-year CVD risk score among black African adults.

## Methods

### Study design and setting

This is an ancilary study that was implemented as part of an ongoing cohort study, the Prospective Urban and Rural Epidemiology (PURE) study in Cape Town, South Africa. The PURE study methodology, study population and setting have been described previously [32, 33].

### Sampling and data collection

A total of 963 participants were interviewed using interviewer-administered structured questionnaires. Body shape questionnaire (BSQ) [34, 36] was utilised to ascertain the level of body image perceptions. The questionnaire also obtained information on the CVD risk factors and exposures. Medical history, blood pressure, height were also recorded.

### Body image and its measurement

This study used pictorial and narrative constructs which was validated amongst South African adults by Mciza and colleagues, to describe body size/image perception and weight estimations [35, 36] respectively. Discordance in weight status, and body size dissatisfaction are two body image components considered for this study.

We determined *weight estimation*, using underestimation or overestimation statuses. To measure i) ‘Feel’ and ‘Actual’ weight Difference (FAD), each participant’s measured weight (using BMI) was compared with ‘feel’ weight with the corresponding silhouettes’ and weight categories [34, 37]. FAD < 0 indicates an increase in the discrepancy between the ‘actual’ and ‘feel’ body weight (i.e. weight underestimation). On the other hand, FAD > 0 means an over-estimation of weight. The discordant weight was also presented as a combination of weight underestimation and overestimation. FAD was used to assess body weight and appearance in previous studies [38, 39].

In addition to weight estimation, we measured size dissatisfaction using ‘Feel-Ideal’ body size difference (FID). Previous studies had reported that increasing FID score indicates an increasing tendency for body size dissatisfaction [40]. FID scores was categorized into three parts viz. FID > 0 (i.e. ‘my size is too large’; FID < 0 (i.e. ‘my size is too small’), and FID = 0 (i.e. ‘I’m satisfied with my size’.

### Cardiovascular disease (CVD) risk score evaluation

We calculate 10-year absolute CVD risk scores for each participant using Framingham Risk Score (FRS) the sex-specific Eqs [26, 41]. The variables in the equations are sex, systolic blood pressure, BMI, age, diabetes, treatment status for hypertension and diabetes. A ‘high CVD risk was considered as a risk scored ≥ 20%.

### Data analysis

The analyses were restricted to the 920 PURE cohort participants (sub-sample) with no known CVD event; 43 participants with known CVDs were excluded. This representative sub-sample utilized for this study was approximately 75% of the existing PURE study cohort participants. Descriptive and bivariate analyses were undertaken. Proportions (in frequencies), means and standard deviations (SD) were presented. Gender-specific correlations between body image perceptions and relative weight change per year (4.5-year period) were determine using scatter plots. Student t-tests were used to compare the associations between selected modifiable CVD risk factors and body image components by obesity status. The difference in body weight at baseline and follow-up divided by the number of years of follow-up was used to ascertain the relative weight change/year.

In addition, one-way ANOVA and t-tests were used to determine sex-specific differences in body image and weight change. Participants’ body image, adiposity, weight, and CVD risk profiles by sex and location were also determined. Normality of the continuous measures was ascertained before applying t-tests. Further, the relationship between body image components and CVD risk scores (at baseline and follow-up), and perceived body weight were determined using mean comparison (ANOVA test) and controlling for demographic factors (age, gender and location). To address our study focus, we also ascertaining how body image components (discordant weight, size dissatisfaction) comparativdely aligned with known risk factors such as smoking, hypertension, systolic blood pressure, diabetes, as well as perceive CVD threat at follow-up (i.e. when body image data was collected). *P*-value (< 0.05) at 95% confidence interval (CI) was used as the statistically significant level.

## Results

### Participants’ characteristics, adiposity and CVD risk profiles

Aspects of the study participants characteristics were described in a previous paper [9]. In summary, the majority of the study participants (*n* = 709, 77%) were women, and from the urban township (*n* = 540, 58.8%); 51% attended any higher school, and 4% had tertiary education. Their mean age was 55.8 years. Only 19% of the study sample had some form of employment, with 78% reported a monthly income of less than R2000 ($121). There were significant differences in age, employment and marital status by gender.

### Adiposity, CVD risk and body image profiles

Data from the follow-up including the adiposity levels, CVD risk and body image profiles of participants by gender and location are presented in Table 1. Majority of the women (84.0%) and over a third of the men had excess adiposity– central obesity (Waist circumference, WC), BMI-based obesity, and or body fat percent (BF%). Similar trends of adiposity proportions were seen by location. The proportions with excess adiposity (obesity/overweight) based on BMI, BF% and WC were 32.2%, 44.3%, and 34.3% for men, and 84.1%, 79.6% and 89.5% for women respectively. Urban men and women had higher mean weight, BMI and BF% compared to their rural counterparts, whereas the rural women had higher median WC than their urban counterparts.

**Table 1:** Adiposity, CVD risk and body image profiles of the study participants at (follow-up) by sex^a^ and location^a^

	ALL		Sex		Rural		Urban	
	*n* (%)	95% CI	Men *n* = 211	Women *n* = 709	Men *n* = 81	Women *n* = 299	Men *n* = 130	Women *n* = 410
**Body weight/Adiposity Levels** (at Follow-up)								
Body weight (mean, SD), kg	78.7 (21.3)	77.4–80.1	69.2 (16.5)	81.6 (21.3)	64.7 (16)	78.5 (20.2)	73.1 (18.4)	83.8 (23.0)
BF%, mean (SD)	39.2 (12.0)	38.3–39.8	25.0 (12.3)	43.3(9.4)	26.2 (8.5	45.0 (8.4)	26.0(12.8)	26.2 (8.5
WC, median (SD), cm	98.9 (19.5)	97.9-101.2	88.2 (17.5)	102.0 (20.5)	90.6 (13.8)	103.6 (17.7)	86.8 (19.2)	100.8 (22.3)
Obesity/Overweight (BMI ≥ 25 kg/m^2^)	664 (72.1)	69.3–75.1	68 (32.2)	596 (84.1)	22 (42.6)**	251 (84.0)**	25 (34.6)**	346 (84.4)**
**CVD Risk Profile**								
Age (mean, SD)	55.8 (10)	55.1–56.5	54.5 (11)	56.2 (10)	56.3 (11)	56.7 (10)	53.3 (10)*	55.8 (10)*
Treated for BP or diagnosed with Hypertension, *n* (%)	382 (41.5)	38.3–44.7	51 (24.2)	331 (46.7)*	16 (19.8)	131 (43.8)**	35 (26.9)	200 (48.8)**
Smoked tobacco, *n* (%)	259 (28.2)	29.1–35.1	123 (58.3)**	136 (19.2)	43 (53.1)**	13 (4.3)	80 (61.5)**	123 (30.0)
Diabetes mellitus status, *n* (%)	129 (14.0)	11.8–16.3	18 (8.5)	111 (15.7)*	3 (3.7)*	37 (12.4)*	15 (11.5)	74 (18.0)
Systolic BP (mean, SD)	140.1 (25)	138.4-141.8	137.2 (25.0)	141.3 (24.4)	133.4 (22.3)*	139.2 (25.8)*	138.5 (24.5)**	142.9 (24.2)**
BMI (mean, SD)	31.0 (8.8)	30.7–32.2	25.4 (5.0)**	33.8 (7.5)**	23.5 (5)*	31.8 (8)*	25.0 (6)**	33.9 (9)**
**Absolute CVD risk**								
10-year CVD Risk Score (mean, SD)	18.7 (12.3)	18.9–19.5	25.2 (13)**	16.7 (11)	24.3 (14)**	15.0 (10)	25.8 (13)**	18.0 (11)
10-year CVD Risk Score ≥ 20%; *n* (%)	304 (33.0)	30.0-36.1	123 (58.3)**	181 (25.5)	43 (53.1) **	60 (20.1)	80 (61.5) **	121 (29.5)
**Body Image components**								
Weight discordance^a^, %	676 (73.5)	57.2–89.8	119 (56.4)	557 (78.6)**	49 (60.5)	232 (77.6)	70 (53.8)	325 (79.3)
Body Size dissatisfaction ^b^ (FID > 0)	290 (31.5)	16.2–40.3	31 (14.7)	259 (36.5)**	9 (11.1)	89 (29.1)**	22 (16.9)	172 (42.0)**

^**a**^ Mean differences between this variable and continuous variables were tested using t-test, and that of categorical variables by Chi-square test;

*** ** *p*-value = 0.001; *p-value = 0.01

^’n’ is count, ‘%’ is frequency; ^**a**^ Discordance (underestimation) in actual and perceive body weight ^**b**^ Feel-Ideal Difference (FID) < 0

The CVD risk profiles indicated that a substantial proportion of women in both the rural and urban communities compared to the men were diagnosed with hypertension (48% vs. 24%), and diabetes (16% vs. 9%), whereas a greater proportion of men than women in the rural (53% vs. 4%) and urban (62% vs. 30%) had smoked tobacco. The mean systolic blood pressure (SBP) was 140 mm Hg, and women had slightly higher SBP than the men (141 vs. 137 mm Hg). The mean 10-year CVD Risk score for the study was 18.7%, and the proportion with CVD risk scores ≥ 20% (i.e. high-risk score) was 33%. Men were at higher risk than the women in both the rural (53% vs. 20%) and urban (62% vs. 30%) communities, and these comparisons were statistically significant (*p* < 0.05).

The proportion with discordant weight was 73.5%, significantly higher among women, and about a third (31.5%) of the study sample were dissatisfied with their body sizes. There were significant differences in body size dissatisfaction by gender in the rural and urban sites.

### Patterns of CVD risk score and body image dimensions

Expectedly, the mean CVD risk score increases with age in both men and women, whereas proportions with weight underestimation decreases with age for men, and remain high (> 74%) for women. Based on the pre-set three age categories, the proportions of those who underestimated their own body weigh decreases (54%-38%) whereas those who were satisfied with current body size apparently remained high (mean, 53%) and unchanged with the age categories (Table 2). A higher proportion of men (84%) and women (40%) aged 60 years and above had risk scores ≥20%. Expectedly, on the overall, 58% of the men and 26% of women had a CVD risk Score ≥20%. Nearly two in five (45%) women had a moderate CVD risk score (i.e. 10.0-19.9%) compared to three in every 10 men. Another important finding is the proportions with a ‘high’ CVD risk Score (≥20%) increase exponentially with age. Within the three age categories considered, the proportion with high-risk increases from 20 to 84% in men, and from 2 to 24% for women. There were also high discrepancies in perception of body size and weight status. The majority of the women (76%) and 49% of men underestimated their weight. Between 15% of men and 37% of women were dissatisfied with their body sizes.

**Table 2:** Patterns of total CVD risk score by body image categories

MEN	Age Category (in years)						Total	
	34–45		46–59		60+			
		**S.E**		**S.E**		**S.E**		95% CI
Framingham CVD risk*	*n* = 56		*n* = 82		*n* = 73		*n* = 211	
Mean, SE	14.1**	1.00	25.1**	1.1	33.9**	1.7	25.2**	23.4–27.1
Moderate Risk Score (10-19.99%), %	46.4**	0.55	31.7**	0.44	16.4**	0.41	30.3**	24.1–36.5
High Risk Score (≥20%), %	19.6**	1.17	62.2**	1.07	83.6**	1.72	58.3**	51.6–64.9
**Body Size Dissatisfaction Status (Proportion**, **%)**	*n* = 56		*n* = 82		*n* = 73		*n* = 211	
Too small (FID < 0), %	32.1	0.03	32.9	0.41	31.5	0.05	32.2	25.9–38.3
Satisfied (FID = 0), %	51.8	0.05	52.4	0.41	54.8	0.04	53.1	46.3–59.8
Too large (FID > 0), %	16.1	0.04	14.6	0.45	13.7	0.02	14.7	9.9–19.5
	*n* = 56		*n* = 82		*n* = 73		*n* = 211	
**Feel-Actual Difference (FAD) -(Weight Discordance)**								
Underestimated Weight (FAD < 0), %	55.4*	0.04	53.7*	0.41	38.4	0.04	48.8**	42.0-55.6
Accurate Estimation (FAD = 0), %	39.3	0.02	40.2	0.43	50.7	0.03	43.6	36.9–50.3
Overestimated Weight (FAD > 0), %	5.4	0.01	6.1	0.31	11.0	0.01	7.6	4.0-11.1
**WOMEN**								
Framingham CVD risk*	*n* = 121		*n* = 313		*n* = 275		*n* = 709	
Mean, SE	8.16**	0.53	15.30	0.51	22.10	0.76	16.72	15.9–17.6
Moderate Risk Score (10-19.99%), %	31.4**	0.32	46.6	0.20	49.5**	0.17	45.1**	41.5–48.8
High Risk Score (≥20%), %	1.7**	5.60	21.7	0.97	40.4**	1.01	25.5**	22.3–28.7
**Body Size Dissatisfaction (FID)**	*n* = 121		*n* = 313		*n* = 275		*n* = 709	
Too small (Feel-Ideal < 0), %	22.3	0.02	23.6	0.03	18.9	0.03	21.6	18.6–24.6
Satisfied (Feel-Ideal = 0), %	38.0	0.02	43.5	0.03	41.8	0.02	41.9	38.3–45.5
Too large (Feel-Ideal > 1), %	39.7	0.02	32.9	0.02	39.3	0.01	36.5	33.0-40.1
**Feel-Actual Difference (FAD)– (Weight Discordance)**	*n* = 121		*n* = 313		*n* = 275		*n* = 709	
Underestimated Weight (FAD < 0), %	74.4	0.03	75.1	0.03	76.4*	0.04	75.5*	72.3–78.6
Estimate accurately (FAD = 0), %	24.0	0.02	22.7	0.02	18.9	0.03	21.4	18.4–24.5
Overestimated Weight (FAD > 0), %	1.7	0.01	2.2	0.01	4.7	0.02	3.1	1.8–4.4

^**1**^**FID– an index to assess body image (dis)satisfaction** [36] ^**2**^**FAD– and index used to assess weight discordance (under**, **over-estimation of**

**weight)** [47]

*** Total CVD risk scores based on Framingham non-laboratory based risk scores; ** *p*-value > 0.001. * *p*-value,>0.01

Comparison of variables by age obtained by ANOVA and chi-square tests

N/B: S.E.M express the variation from the population mean, and therefore explains how the mean in a group is closer to the population mean

^**^**^There were no significant difference in FID or FAD and age categories in men and women

### Body image and relative weight change

**Fig. 1:**
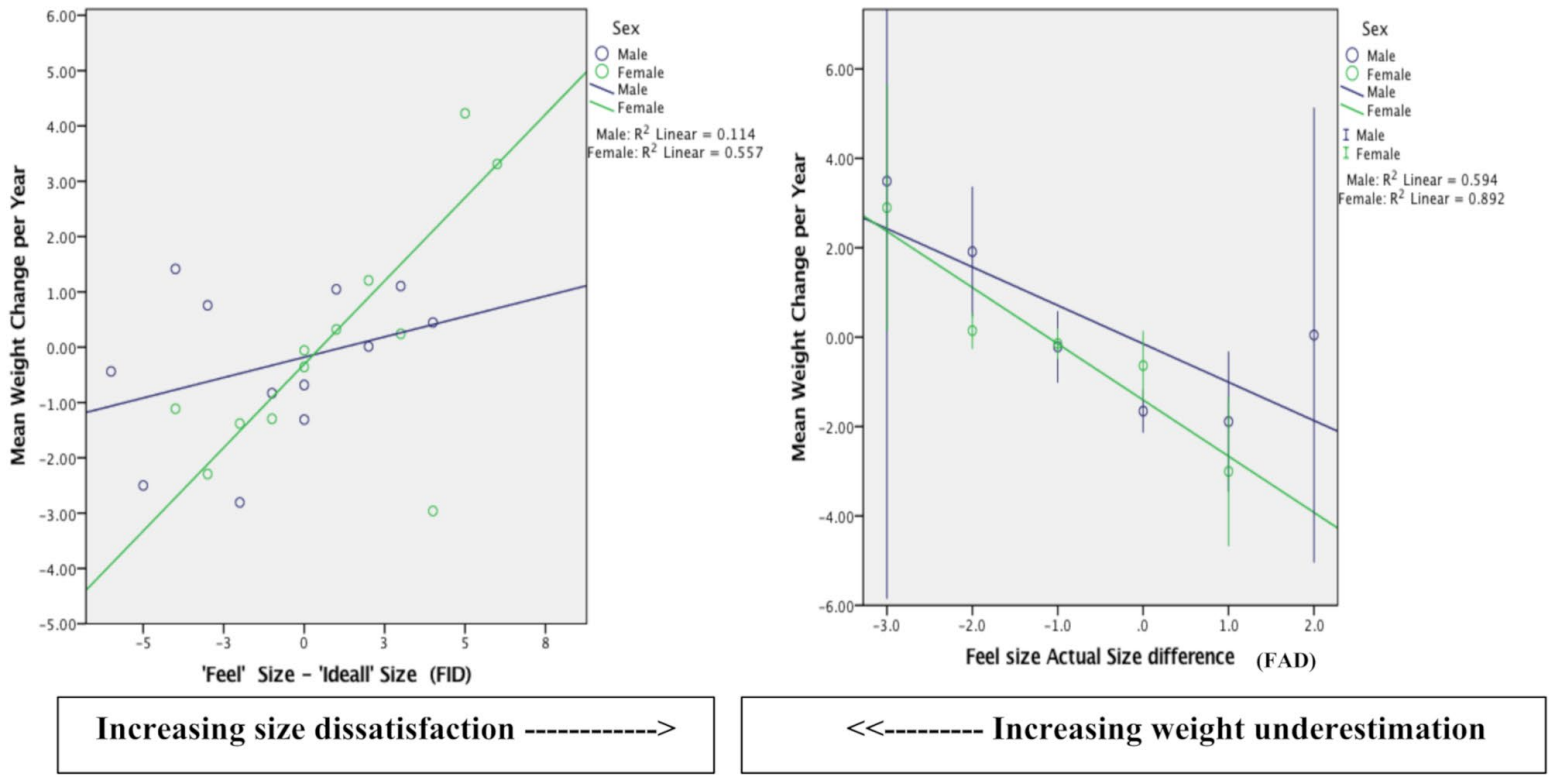
Box plot showing the relationship between body image (FID and FAD) and change in weight (in kg) per year by gender

The sex-specific differences in body image and weight change are shown in Fig. 1. FAD (increasing weight underestimation) was associated with relative weight gain (change/year). FID (increasing size satisfaction) was also positively associated with a significant gain in relative weight change/year (i.e. weight gain). Specifically, increasing weight underestimation (i.e. as FAD > 0) led to increased weight gain among men and women. In the women group, a weight gain > 2.0 kg/year was seen with FID index ≥5, whereas a weight loss of > 3.0 kg/year was seen with FAD index ≥2.

### Comparison of CVD risk factors and body image in overweight/obese and normal weight persons

**Fig. 2:**
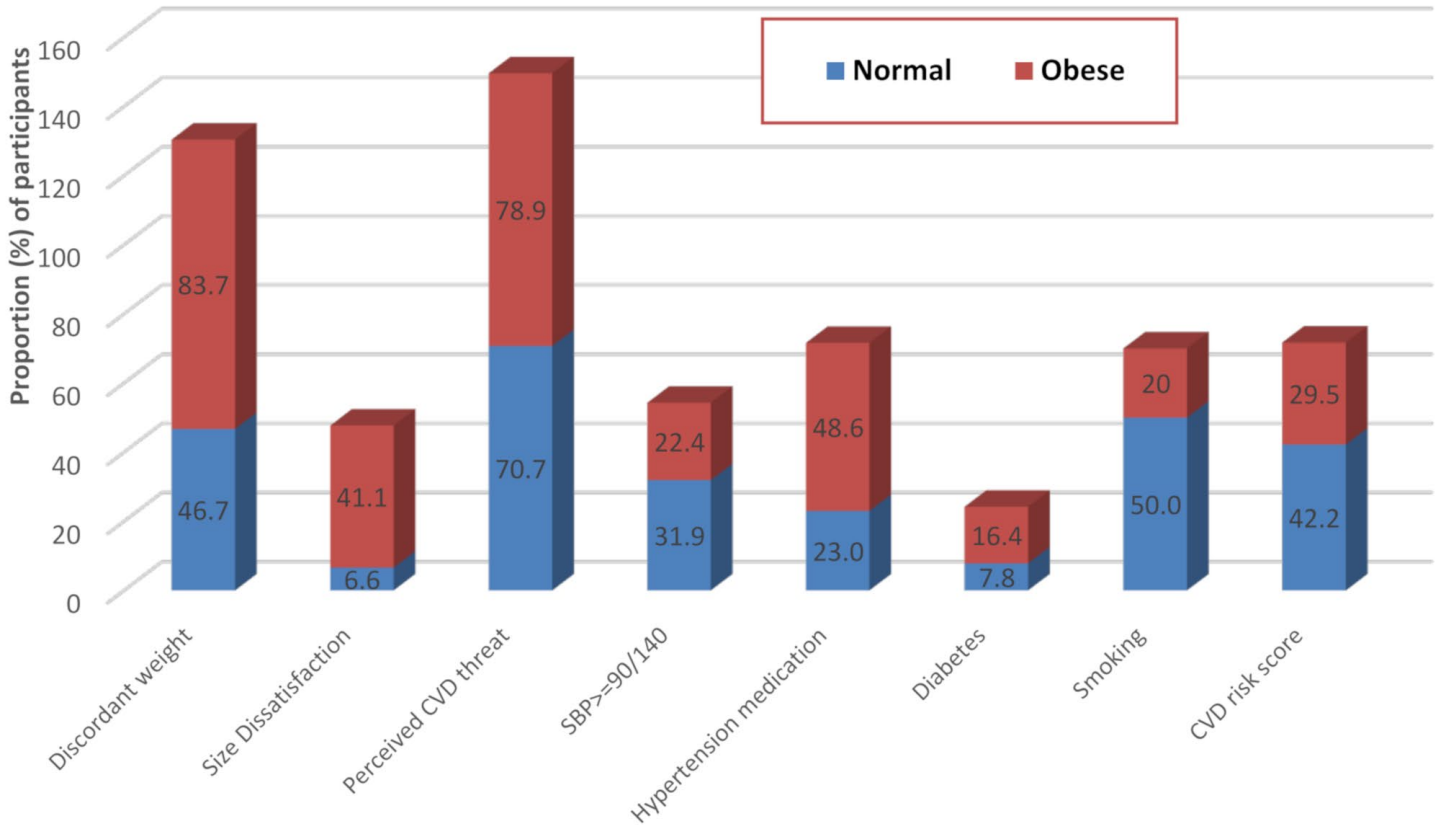
Comparison of modifiable CVD risk factors and body image components by obesity status (at follow-up)

A comparison of the modifiable CVD risk factors and body image components by overweight/obesity status (BMI > 25 kg/m^2^) and the normal weight (BMI < 25 kg/m^2^) is presented in Fig. 2. Unexpectedly, a higher proportion of normal weight individuals compared to obese (42% vs. 29%) had a total 10-year CVD risk scores > 20%. This disparity could, perhaps, be due to a the fact that a significantly higher proportion of persons with normal body weight (compared to the obese group) were smokers (50% vs. 20%), had confirmed hypertension status (49% vs.23%) and had diabetes (16% vs. 8%). The chi-square tests showed significant differences (*p*-value < 0.01) between obese and normal weight for all the listed variables (in Fig. 2).

### Mean comparison of CVD risk score with body image

In Table 3, The mean comparison of CVD risk scores (at baseline and follow-up) using ANOVA test showed a somewhat weak but significant association with weight estimation (underestimation) at baseline (*p* = 0.051) and follow-up (*p* = 0.049). In addition, perceived body image satisfaction had a moderately strong association with cardiovascular disease risk score only at baseline.

**Table 3:** Mean comparison of CVD risk scores with body image and weight perception parameters

		CVD Risk Score (Baseline)	CVD Risk Score (Follow-up)
**Body Image Satisfaction**			
I’m ‘Too Small’	Mean	22.6653	18.85
	Std/SEM*	11.7/0.77	12.6/0.83
I’m ‘Satisfied’	Mean	21.54	18.65
	Std/SEM*	11.1/0.50	12.6/0.61
I’m ‘Too large’	Mean	20.53	18.89
	Std/SEM*	10.6/0.61	12.3/0.70
*p*-value		**0.028**	0.949
**Body Weight Estimation**			
	Mean	26.21	25.77
Underestimation	Std/SEM*	09.3/0.87	13.0/0.99
	Mean	15.66	17.64
Accurate Estimation	Std/SEM*	10.24/0.46	12.5/0.51
	Mean	19.33	19.22
Overestimation	Std/SEM*	10.4/0.76	12.0/0.88
*p*-value		**0.051**	**0.049**

*Std (Standard deviation); **SEM**– Standard error of mean

## Discussions

### High adiposity and CVD risk profiles

At follow-up, the majority of the study sample had excessive adiposity proportions in both rural and rural communities, and these ranged between 80% and 90% in women, and 32% and 44% in men. A similar trend in women (82-96%) and men (26-62%) were reported in this same study population based on baseline data in 2010 [33]. These trends indicate a sustained excessive adiposity rates in this population.

Compared to the previous studies in South Africa and elsewhere, findings from this study indicate a CVD risk profile with a high prevalence of tobacco smoking (28%), reported diabetes (14%), mean systolic BP (140 mmHg) and BMI (30.6 m^2^/kg), raised body fat (38%) [25, 26]. A constellation of cardiometabolic syndromes can be expected as there is high prevalence of obesity, tobacco use, and increasing hypertension reported among the black South Africans. Findings from country-wide South African population study involving 13 cross-sectional studies conducted with 14,772 adults had reported lower mean CVD mortality risk with approximately 18% considered as high risk [25]. In that study, a high proportion (33%) of the study population were also considered as ‘high risk’ with a 10-year mortality risk ≥ 20% [25], particularly among the obese. In addition, another study conducted in Cape Town black communities by Peers and her colleagues [26] reported a lower mean CVD risk score of 11.1% in men, and 6.8% in women and only 13% of men and 7% of women had CVD risk ≥ 20%.

Our study, in contrast, reported a higher mean 10-year CVD risk score of 25.2% in men and 16.7% in women. Predominantly overweight and normal weight participants had high CVD risk score (> 20%), with half (∼ 50%) of the women with moderate CVD risk scores (10–20%). The proportions of persons with CVD risk ≥ 20% was also comparatively higher in men (58.3%) compared to women (25.5%). Also, those with normal body weight compared to those with obesity (42% vs. 30%) had a high CVD risk score (i.e. total 10-year CVD risk scores > 20%), due partly to their body image perception and adverse CVD risk profiles. Our study findings indicates that there is a high CVD risk in men and women in these communities, as a third of the study sample were at high risk of CVD deaths– higher than previously recorded [42].

### Body image and changes in weight and adiposity

Increasing weight overestimation (i.e. weight discordance) was associated with a decrease in weight/year. In contrast, the increase in body size dissatisfaction was associated with a significant gain in weight/year. This finding might suggest that those who overestimated their weight could, after self-evaluation develop self-efficacy and a willingness over time to lose weight. In addition, this could be probably due to cultural norms (favouring large body size), emotional eating, and or access to cheap unhealthy food. Previous studies had reported weight overestimation resulted in weight gain [43, 44]. On the other hand, weight tended to increase, unexpectedly, as people were dissatisfied with their body sizes, indicating that other factors (such as the influence of culture, socio-economic, and genetics, etc.) outside of body image perceptions, and personal attitudes may play some role. Notably, weight gain was attributed to increasing body size dissatisfaction in the women compared to men, whereas weight loss in women as well as in men was linked with increasing weight discordance. Moreover, findings from two studies (qualitative and quantitative) involving the same study (black South African) population, showed that body size dissatisfaction with perceived risk was linked to the willingness to lose weight among the predominantly obese populations who perceived some threat of obesity and CVD [9, 16]. Connecting the earlier findings with the current one, it could mean that though body image dissatisfaction can lead to intention to lose weight, it might not result in actual weight loss over time.

### Weight discordance and body size dissatisfaction status, and CVD risk score

Weight discordance was highest among the overweight and obese persons compared to the normal weight, and this was also the case for body size dissatisfaction. Importantly, overestimating own body weight was associated with relative weight loss in this study sample, indicating a probable challenge in the prevention of obesity and overweight in these settings. In addition, a considerably high proportion of predominantly overweight participants had high CVD risk score (> 20%), and most of them had also underestimated their weight. These findings speculatively indicates higher cardiovascular risk propensity among persons with obesity and body image discrepancies. It could be explained that having strong concerns about weight can lead to behaviours or lifestyle that can impact on CVD risk factors. Peer et al., in her study on black South Africans, had shown that the obese group had a comparatively high proportion of all the modifiable CVD risk factors, except for smoking, and form more than half the number that was at high CVD risk [42]. The data on Fig. 2 indicates that more than half of the participants in our study who had smoked were at high CVD risk. These findings have implications for community-based obesity prevention.

### Implications for integrated obesity prevention

In this study and in other literature, body image, particularly, weight underestimation is associated with relative weight gain in black Africans, as it engenders inactivity, adverse eating behaviour and overweight tendencies [9, 11, 16]. In addition, our study showed that high weight discordance and large body size preference to be independently associated with weight change and high CVD risk score amongst black African adults with obesity. Also, in this study population, men and women had adverse CVD risk profiles. We also shown that those who underestimated their body weights (i.e. decreasing weight overestimation) had a significant weight change (loss) over time.

The above findings, can be an important yardstick to consider in targeting body image interventions in this reference population. Underestimating one’s own weight could indicate a tendency to not support personal weight loss intervention. A study among young adults showed that weight underestimation was significantly associated with inappropriate weight control practice. On the other hand, inappropriate body image dissatisfaction have been shown to hinder the adoption of personalized weight-loss intervention among women in another township located near Cape Town [45]. In the previous study with the same study population, body size dissatisfaction with perceived obesity threat was linked to willingness to lose weight [16]. Also even though the CVD risk is high, there was the low perception of CVD threat among black overweight adults, particularly [16]. However, in another non-African population also, body images perceptions has been associated with metabolic syndrome, and CVD risk factors [13]. This would mean that, in a black population (and other non-African population) with an inherent problem of high obesity and negative body image perceptions, inadequate perception of obesity and CVD risk could hinder personal health promotion behaviours. This has implications for community-based obesity prevention initiatives. This calls for a concerted effort to develop cost-effective participatory community-driven health promotion interventions tailored to addressing obesity epidemic and rising CVD risk and morbidly, considering weight perception issues. Importantly, the effect of obesity risk perception on weight control behaviour cannot be overlooked in settings with dominant weight underestimation and high obesity prevalence. An integrated obesity prevention programme targeting black South Africans should consider a sustained healthy weight maintenance intervention focusing on personalized self-assessments of weight gain intensions and body size preferences.

### Strengths and limitations

This is important, as it uses measured and perceptive measures to clearly show that body size perception and weight discordance are associated with changes in weight overtime, and mean CVD risk score in South African adult population. The study has some limitations, however. The parameters used in calculating absolute CVD risk such as diabetes and tobacco smoking were self-reported and might have been under-reported. This applies also to body image parameters– as these were not measured with modern computer-based objective techniques. In addition, the study participants were recruited from existing cohorts in two selected black communities in two provinces, and may not be a true representation of the entire South African population. The effect of self-reported data and sample representativeness could also have impacted on the results. We also believe that confounders such as HIV/AIDS or TB, and weight trajectory might have impacted on body image perception and weight change and could have introduced bias of some sort. In addition, a possible selection bias might have been introduced as the study participants were predominantly women, most of them unemployed. This notwithstanding, these findings can be applied to the poor-resource setting of South African and other similar African populations. Similar studies in larger population and many settings should be undertaken to further validate the relationship between body image index and CVD risk scores controlling for other exposures and modifiable risk factors. It is important to use a modern computer-aided body image measurement technique such as Body-Image Assessment Software that can help assess body image dissatisfaction or weight underestimation more objectively [46].

## Conclusion/Recommendations

Increasing weight discordance, particularly weight underestimation was associated with weight gain had a weak association with total 10-year cardiovascular risk scores in the study population.

Interventions to reduce obesity and mitigate CVD mortality risk should address the discrepancies in weight perception, poor obesity risk perception, and personal motivation towards weight loss reported previously in this study population [16]. Obesity and CVD risk prevention programmes targeting black South Africans should consider a sustained healthy weight maintenance intervention focusing on personalized self-assessments of weight gain intensions and body size preferences. We, therefore, recommend that community-driven intervention that supports the right messaging on the possible benefits of assessing one’s own body size and body weight using simple measuring tape and weight scale can be co-developed with community members, implemented and evaluated. In addition, developing and implementing culturally acceptable body image assessment interventions can help identify high-risk individuals with body image discordance in the community for brief behaviour change communication. Importantly, tailored co-developed digital health interventions that could incorporate body image assessment and self-evaluation can be an innovative strategy to support health promotion in settings with high prevalence of obesity and cultural perceptions of body size.

## Data Availability

All the data are contained within the manuscript and are available from the corresponding author upon reasonable request.

## Abbreviations

BMI: Body mass index
BF%: Body Fat Percent
BP: Blood Pressure
BSQ: Body shape questionnaire
CVD: Cardiovascular Disease
FAD: Feel minus Actual Weight Difference
FID: Feel minus Ideal body Image Difference
PURE: Prospective Urban and Rural Epidemiology
WC: Waist Circumference
